# Magnetic resonance evaluation in long term follow up of operated lateral tibial plateau fractures

**DOI:** 10.1186/s12891-015-0633-z

**Published:** 2015-07-26

**Authors:** Georg Mattiassich, Ernst Foltin, Martin Pietsch, Oliver Djahani, Albert Kröpfl, Martin Fischmeister, Georg Scheurecker

**Affiliations:** Trauma Center Unfallkrankenhaus Linz - Teaching Hospital of the Paracelsus Medical University Salzburg, Garnisonstrasse 7, Linz, 4010 Austria; Ludwig-Boltzmann Institute for Experimental and Clinical Traumatology, Vienna, Austria; Private Practice for Trauma Surgery, Schlierbach, Austria; Department of Orthopaedic Surgery, General and Orthopaedic Hospital Stolzalpe, Stolzalpe, Austria; Department of Trauma Surgery, Diakonissen Clinic Schladming, Schladming, Austria; Institute for CT- and MRI-Diagnostic at Schillerpark, Linz, Austria

**Keywords:** Tibial condylar fracture, Tibial plateau fracture, Magnetic resonance imaging, Long-term results, Whole-organ magnetic resonance score (WORMS)

## Abstract

**Background:**

A lack of data exists on the long-term magnetic resonance imaging (MRI) findings after surgical repair of tibial plateau fractures (TPFs). We evaluated the MRI findings 13 to 31 years after surgical repair of TPFs, focusing especially on the pathological changes in the ligaments, menisci, and cartilage.

**Methods:**

Twenty-three patients with 24 TPFs underwent open reduction and internal fixation with the same fork-shaped surgical plate that was used in our institution until 1999. No patient underwent preoperative or immediately postoperative MRI. The knees of all patients who underwent plate removal were examined by axial, coronal, and sagittal MRI. The Knee Injury and Osteoarthritis Outcome Score (KOOS) and whole-organ magnetic resonance score (WORMS) were determined in all patients.

**Results:**

All 24 knees exhibited MRI abnormalities. An unexpectedly high number of pathological changes in the menisci and ligaments were observed. No meniscal or ligamentous injuries were documented at the time of the injury or initial surgery, but meniscal injuries manifested in the long term. MRI in almost all cases showed a damage to the lateral meniscal, the severity of which was related to the degree of tibial plateau widening, but not to the severity of the lateral joint surface impression. The overall condition of the knee joint was satisfactory as measured by the WORMS, and there was a weak correlation between WORMS and KOOS.

## Background

To our knowledge no studies have reported magnetic resonance imaging (MRI) findings more than 10 years after surgical repair of tibial plateau fractures (TPFs). Therefore, we evaluated the MRI findings in conjunction with the clinical changes that occurred after a long-term follow-up of 13 to 31 years following surgical repair of TPFs.

The objective of this study was to use these long-term MRI findings to describe the periarticular pathological changes in the ligaments, menisci, and cartilage after surgical repair of TPFs [1–4].

## Methods

### Patients

**Fig. 1:**
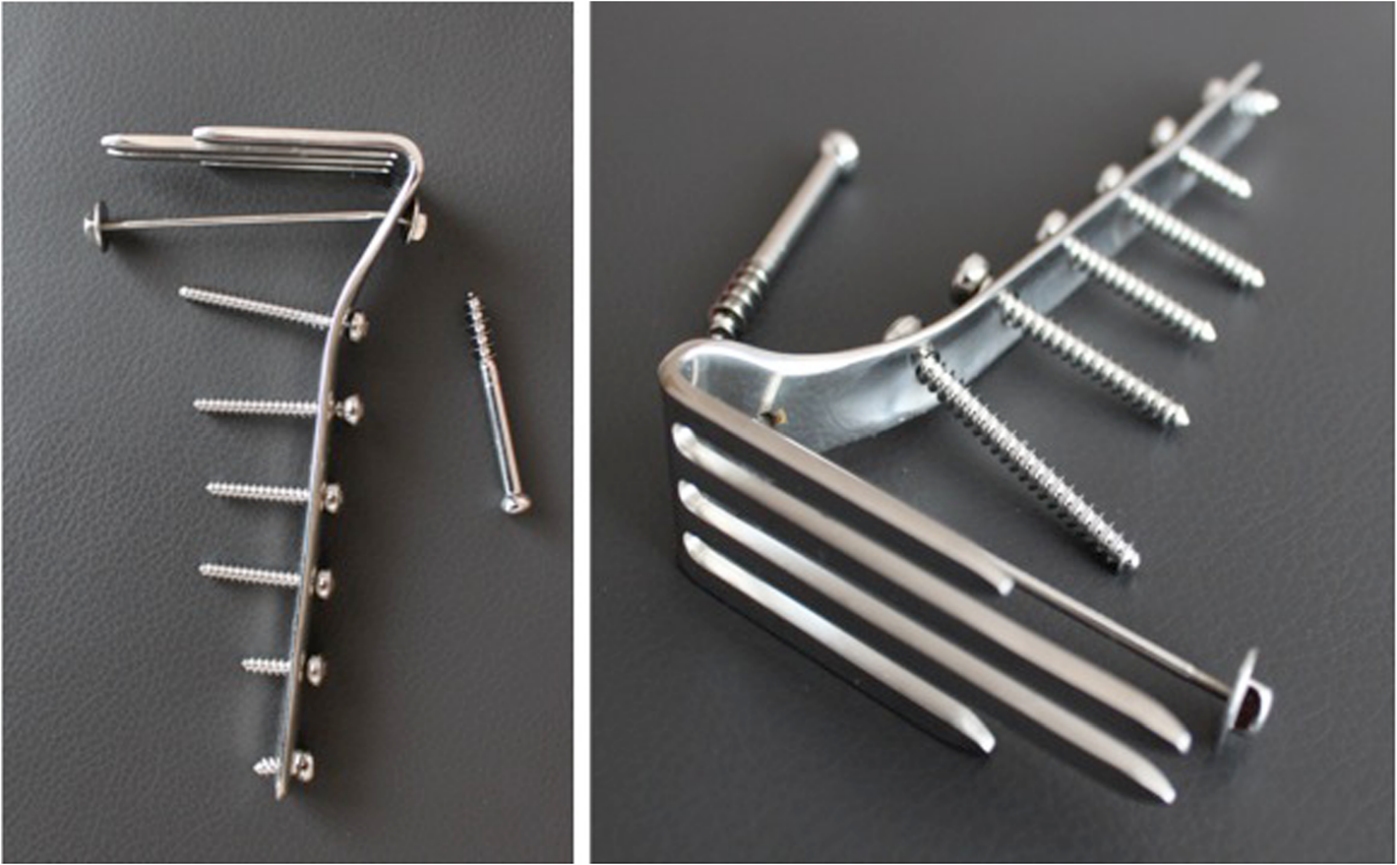
Fork-shaped plate

Twenty-three patients undergoing open reduction and internal fixation for 24 TPFs (displaced B- and C-type fractures according to the AO/ASIF classification) were treated with the same fork-shaped plate (Ulrich GmbH & Co. KG, Ulm, Germany) (Fig. 1). This plate was the standard implant at our institution until 1999, at which time it was replaced by anatomically shaped plates with fixed-angle screws. All operations were performed by experienced senior surgeons. The now outdated implant was the subject of two studies in 1985 and 1999 at our institution [5, 6]. These patients were subsequently re-evaluated with a focus on clinical findings and radiological development of osteoarthritis (OA) [7].

At the time of TPF repair, none of the patients were subjected to preoperative or immediately postoperative MRI. Documentation was performed at all stages of treatment from the initial injury to the postoperative phase.

No patient in this study initially required a meniscectomy or ligament tear repair. Injuries to the cruciate ligaments were evaluated intraoperatively by manual traction with a probe and documented in the operation report. All patients were treated according to the same postoperative treatment plan: passive motion was initiated on the first postoperative day, and full weight bearing was not allowed for 12 postoperative weeks. Hardware removal was performed at the earliest one year after index surgery only if bone healing was proven clinically and radiologically.

Patients who participated in these former studies were contacted for re-evaluation. All available patients who had the hardware removed and showed no contraindications for MRI evaluation were included in this study. All operation reports were analyzed with respect to the intraoperative findings, with particular focus on meniscal and ligamentous injuries.

Coincidentally none of the patients showed meniscal injuries or ligament tears at the index surgery after analyzing the operation report.

### Scores and classifications

All patients completed the Knee Injury and Osteoarthritis Outcome Score (KOOS) [8, 9]. The sum of the five KOOS components was used for further analysis.

### MRI acquisition and interpretation

The MRI protocol included the performance of axial, coronal, and sagittal scans with proton density-weighted sequences with and without fat saturation by a 1.5-Tesla MRI unit (Espree; Siemens AG, Erlangen, Germany). All patients were placed in the supine position. The knee was scanned with a dedicated knee coil.

The MRI findings in the injured knee were examined to identify effusion, chondral damage, and changes to the menisci and cruciate and collateral ligaments. The semiquantitative whole-organ magnetic resonance score (WORMS) was assessed by summing all subscores [10].

All MRI scans were reviewed by a radiologist specialized in musculoskeletal and especially in knee MRI (G.S.). The radiologist had no access to any patient information or records, ensuring an unbiased review of the scans. The condition of the menisci was classified as follows: 1, normal; 2, degeneration without rupture; 3, small rupture; 4, rupture or subluxation. Degenerative changes were defined as described by Crues et al. [11] and included all grades of degeneration. Small lesions were defined as zone 3 tears according to Cooper’s classification of circumferential tears [12].

### Radiographic measures

**Fig. 2:**
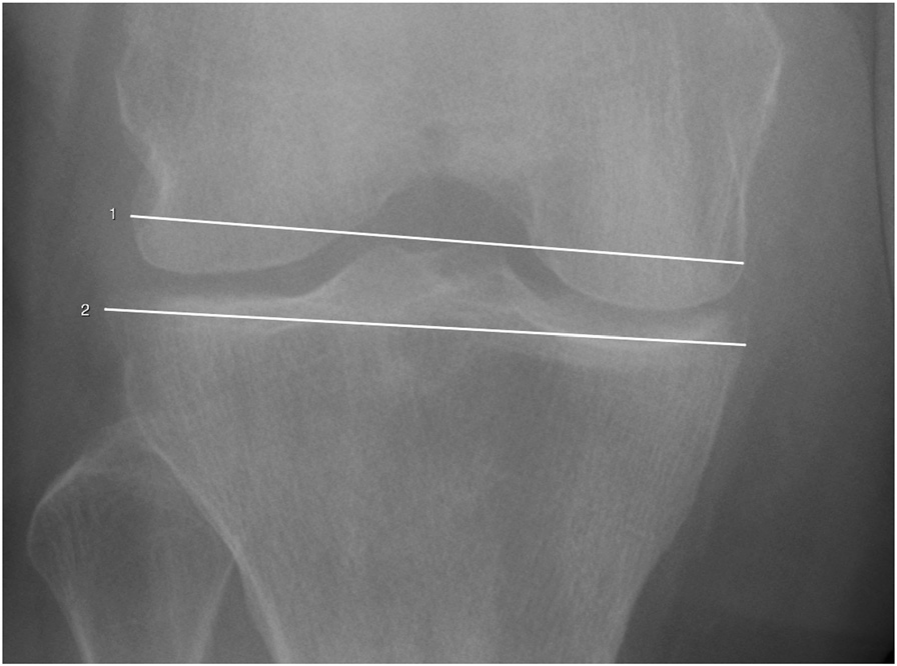
Method of measurements on plain radiograph anteroposterior (right knee – unaffected side): line 1 was drawn between the cortices of the medial and lateral condyle near the articular surface, line 2 was drawn from medial to lateral rim of the proximal tibia (without osteophytes). Tibial widening was given as the quotient of these two parameters and expressed as a percentage of the femoral width

**Fig. 3:**
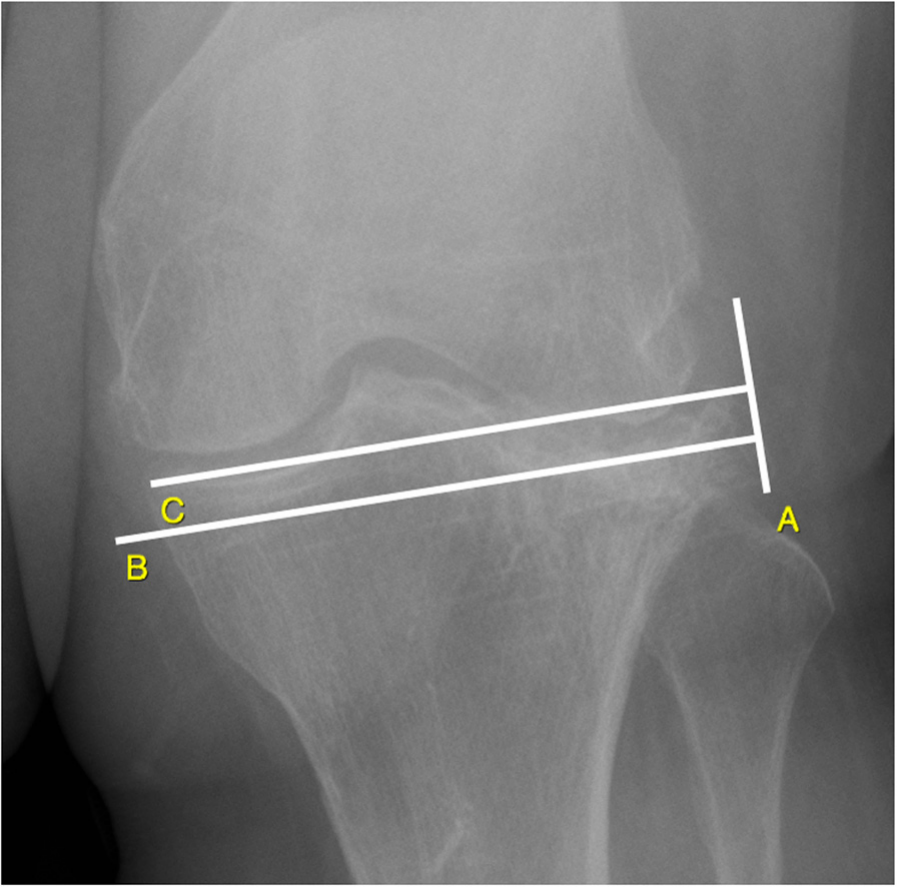
Method of measurements on plain radiograph anteroposterior (left knee – affected side): Lateral plateau impression is the difference between line (**b**) drawn from the maximum impression of tibial plateau baseline parallel to line (**c**) that is drawn in extension of the medial plateau parallel to the joint line. Line (**a**) is the common perpendicular to lines (**b**) and **c**. There was valgus alignment of the knee joint (not shown)

On the anteroposterior radiograph the width of the tibial plateau (TP) was measured from the medial to lateral rim of the proximal tibia. The femoral width was measured as the distance between the cortices of the medial and lateral condyle near the articular surface (Fig. 2). Measurements were done digitally at a workstation by 2 independent orthopedic trauma surgeons. Tibial widening was given as the quotient of these two parameters and expressed as a percentage of the femoral width. The impression depth was measured on the anteroposterior X-ray and was defined as the distal-most point of an articular fragment as measured from the level of the TP (Fig. 3).

### Statistical analysis

Spearman’s rank correlation coefficients (RS) were calculated with 95 % confidence intervals (95 % CI) to evaluate the relationships between variables. Allowance was made for ties. The median value is used throughout the study as the location parameter, and the minimum, lower quartile, upper quartile, and maximum are presented in brackets. Systat 13 (Systat Software, Inc., Chicago, IL) was used for the statistical calculations. The module ‘exact tests’ were used as appropriate.

## Informed consent

The study was approved by the ethics committee of the Austrian social insurance system for occupational risks. All patients were contacted in accordance with the guidelines established by the ethics committee and provided written informed consent after being informed about the protocol and purpose of the study.

## Results

### Patient characteristics

Sixteen male and 7 female patients participated in the study. These 23 patients had 24 TPFs (1 male patient had bilateral simultaneous TPFs). Fourteen AO type B and 10 AO type C fractures were encountered. All patients underwent open reduction and internal fixation using the fork-shaped plate. No patient had an open fracture.

The median (minimum, lower quartile, upper quartile, maximum) age at injury was 40 years (18, 33, 46, 60 years). The follow-up period was 21 years (13, 15, 28, 33 years). The width of the tibial head was 105 % (99 %, 101 %, 109 %, 113 %) of the width of the femoral width. Postoperatively, this value improved to 102 % (94 %, 100 %, 103 %, 108 %); at follow-up, it changed to 100 % (94 %, 98 %, 102 %, 107 %). The injury produced an impression of 10.0 mm (0.0, 3.0, 12.0, 24.0 mm), which was surgically corrected to 0.0 mm (0.0, 0.0, 1.5, 3.0 mm). At follow-up, the impression was 0.0 mm (0.0, 0.0, 3.0, 14.0 mm); thus, some patients exhibited a settling of the lateral tibial condyle.

### Osteoarthritis, Bone marrow edema and osteophytes

**Fig. 4:**
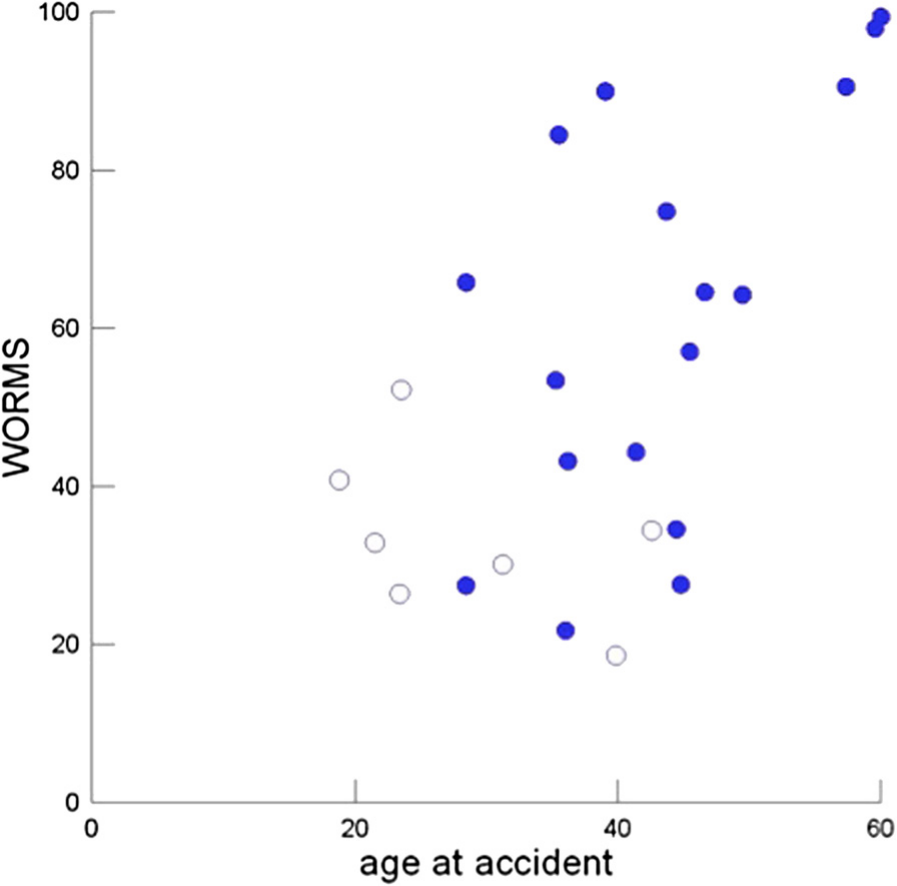
Relation between WORMS and age at time of the accident. Empty circles represent patients with the lowest dislocation at time of accident (Impression less than 10 mm and tibial condylar widening of less then 105 % of the femoral condyles)

The KOOS was 418 points (127, 325, 471, 500 points), and the semiquantitative WORMS was 49 points (21, 31, 76, 96 points). Only a weak association was noted between the subjective complaints as measured by the KOOS and OA as measured by the WORMS: RS = −0.37 (95 % CI, −0.70 to −0.03). A moderate influence of age at the time of trauma on WORMS could be ascertained: RS = 0.57 (95 % CI, 0.25 to 0.88), whereas the duration of time from trauma to follow-up had no effect: RS = −0.01 (95 % CI, −0.40 to 0.38). This influence of age is illustrated in Fig. 4.

**Fig. 5:**
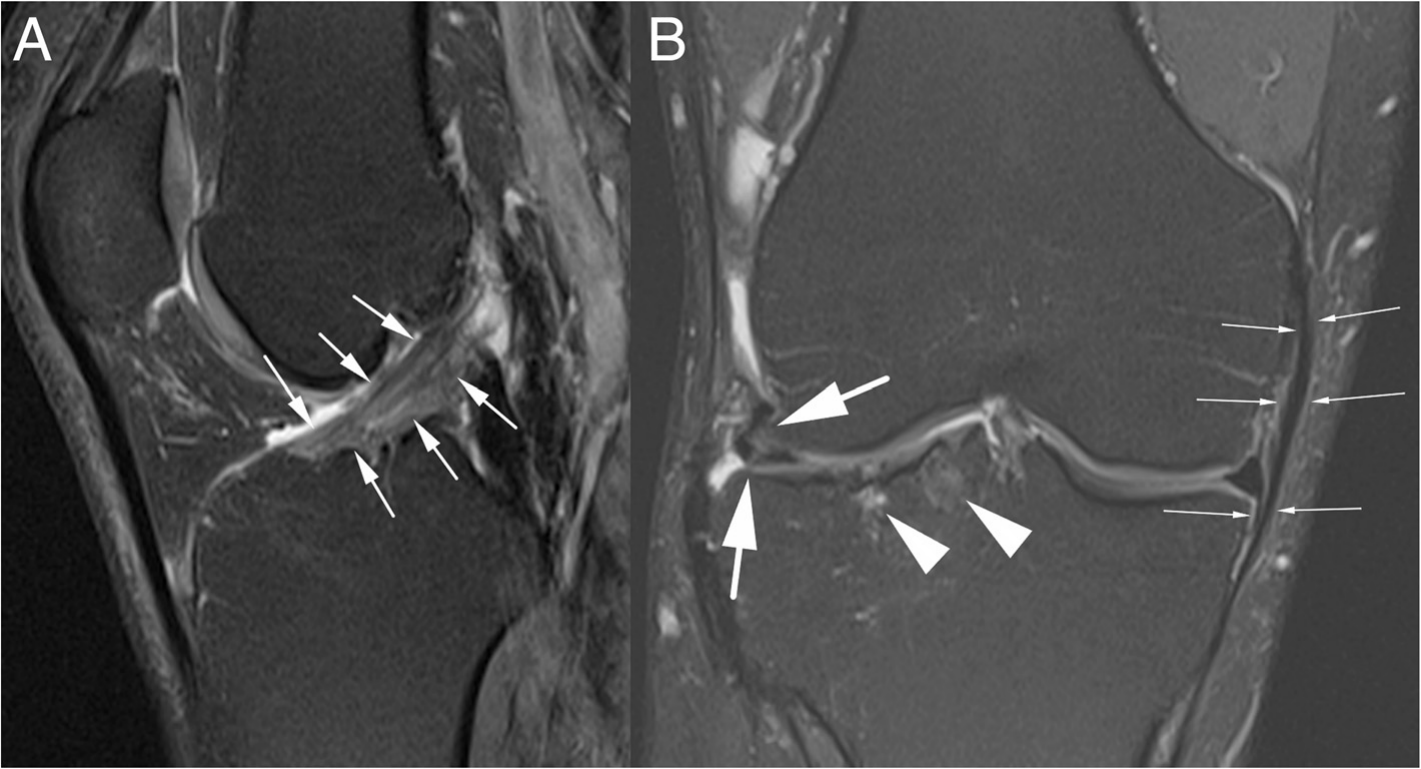
Fat-suppressed proton-density-weighted (FS-PDw) magnetic resonance imaging (MRI). Fig. 5 **a** (sagittal plane) – Signs of degeneration of anterior cruciate ligament with abnormal high signal intensity. Fig. 5 **b** (coronal plane)– Large arrows: Lateral meniscus with degeneration and extrusion (tear not shown). **b** - Arrow heads: Bone marrow edema. **b** - Small arrows: Partial thickening of the medial collateral ligament on MRI

Twenty-three patients showed signs of fluid signal intensity in the bone marrow unrelated to MRI artifacts after metal removal (Fig. 5 b – arrow heads). A bone marrow edema pattern and bone marrow edema-like lesions are signs of OA and are not specific to TPF. The median WORMS subscore for bone marrow edema was 3 points (0, 2, 5, 9 points). There was no correlation with the KOOS: RS = −0.09 (95 % CI, −0.42 to 0.24).

Eight knees had no osteophytes. The WORMS subscore for osteophytes was 5 points (0, 2, 17, 33 points). It was weakly correlated with the KOOS: RS = −0.39 (95 % CI, −0.68 to −0.09).

### Menisci

**Fig. 6:**
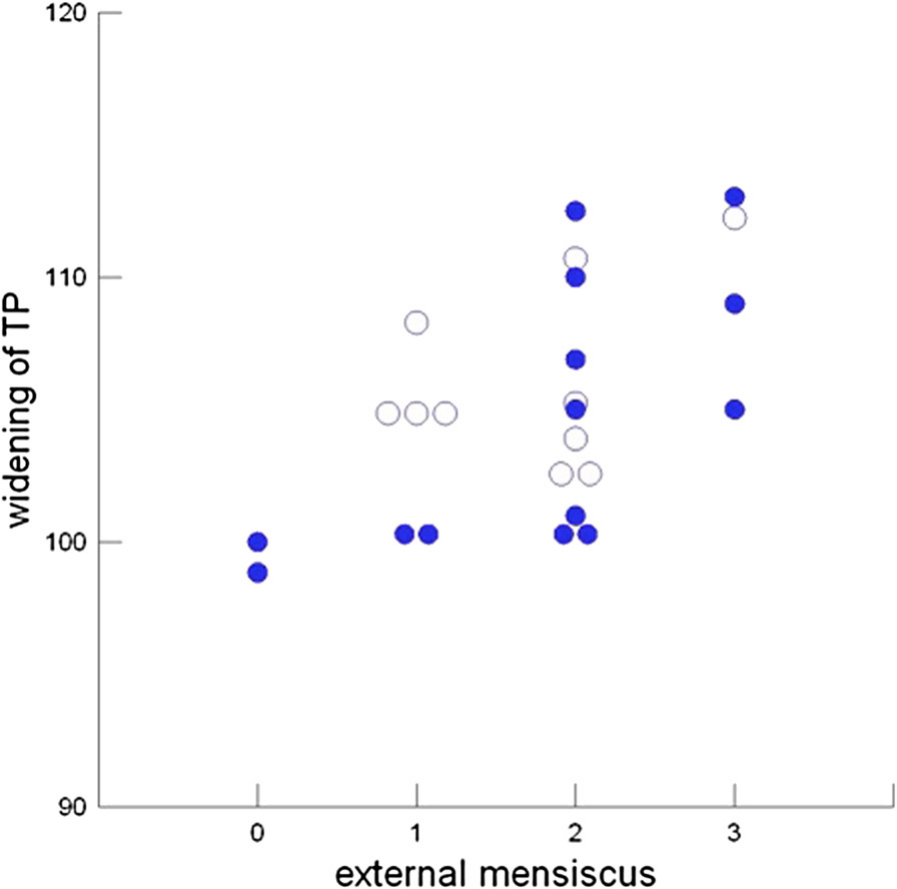
Influence of the widening of the tibial plateau (TP) at the time of the accident on lesion of the external meniscus at the time of FU: full circles denote TCF where the widenig of the TP was corrected by the operation to less then or equal to 102 % of the of the femoral width

Twelve patients had lesions of the anterior (n = 9) and/or posterior (n = 7) root of the medial meniscus as shown by MRI. Two menisci exhibited no lesions; 9 degenerative lesions, 13 showed small ruptures (Fig. 4 b – Large arrows). Twenty-two patients had a lesion of the anterior (n = 19) and/or posterior (n = 16) root of the lateral meniscus. The external meniscus appeared normal by MRI in 2 patients, exhibited degeneration without rupture in 6, exhibited small ruptures in 12, and exhibited a rupture or subluxation in 4. The condition of the lateral meniscus at the long-term follow-up was dependent on widening of the TP at the time of the injury: RS = 0.56 (95 % CI, 0.27 to 0.85). This relationship was maintained when the analysis was restricted to patients in whom the widening of the TP was surgically corrected (i.e., widening of the TP ≤102 %): RS = 0.76 (95 % CI, 0.53–1.00) (Fig. 6). For comparison, the depth of the impression caused by the trauma exhibited no effect on the condition of the external meniscus at the time of follow-up: RS = 0.10 (95 % CI, −0.32 to 0.51). Notably, none of the patients underwent a meniscectomy at the initial operation. Furthermore, no meniscal injuries were documented in the operation reports.

Nineteen patients declared that their walking distance was not limited (24 knees). One patient was unable to walk more than a few steps, two were able to walk for up to 15 min, and two were able to walk for up to an hour. All five of these patients with restricted ambulatory ability had a lesion of the lateral meniscus: three had small tears and two had subluxations. All five patients exhibited a ≥105 % widening of the TP after the injury.

### Collateral and anterior cruciate ligaments

Sixteen of 24 knees exhibited thickening of the medial collateral ligament on MRI (Fig. 4 b – Small arrows). Only six patients had a normal appearing anterior cruciate ligament (ACL) on MRI. Eight had some signs of degeneration, and the ACL was elongated in nine (Fig. 4 a). One ACL was ruptured and underwent a secondary operation for repair of subjective instability. This patient had no clinical instability in the clinical follow-up examination. The subjective feeling of instability was not associated with the ligamentous changes.

## Discussion

The high number of pathological changes to the menisci and ligaments in this small group of patients was unexpected. Delmarter et al. reported the presence of ligament injuries regardless of the fracture pattern in approximately 20 % to 25 % of TPFs [13]. They further proposed that late instability following TPFs is a major cause of unfavorable results. Whether this late instability is caused by ligamentous laxity and/or bony deformity remains debatable. We examined TPFs without meniscal or relevant ligamentous injuries (higher-grade medial or lateral collateral or cruciate ligament injuries) at the time of the index procedure using MRI during a long-term follow-up period of at least 13 years.

### Development of OA

The overall condition of the knee joint as measured by the semiquantitative WORMS showed satisfactory results in our patients. The WORMS provides a good indication of the overall status of the knee joint, expressing the result as a number. The WORMS showed only a weak correlation with the subjective KOOS. The weak correlation between age at the time of the initial trauma and a lower WORMS indicated that younger patients seem to have better outcomes. The time of follow-up did not seem to have a negative influence on the development of OA, although younger patients tended to exhibit less severe injury patterns. This may be because patients in this age group have better bone quality [14]. A different type of TPF is known to occur in patients with osteoporosis: crush-type fractures are more common in patients of advanced age and are more difficult to surgically reconstruct. This could explain why a younger age at the time of trauma is associated with a lower risk of OA [15]. This association was confirmed in the present study. Some degree of OA due to primary cartilage damage is to be expected, even with sufficient surgical management that provides stable osteosynthesis and allows for the performance of exercises in the early postoperative phase [16].

### Ligaments and bone marrow edema

All 24 knees had abnormalities on MRI. All but one knee had at least one focus of fluid signal intensity in the bone marrow, the ACL showed at least degenerative changes in a majority of patients, and two-thirds of the patients had a thickened medial collateral ligament. These changes suggest that alterations of the ligaments are induced by increased stress due to an imperfect fit of the bony structures.

In 1994, Honkonen [17] suggested that repair of a collateral ligament should be considered if there is any mediolateral instability in the extended knee joint after rigid internal fixation. Most of the patients in our study group had stable knees.

### Menisci

The lateral meniscus was preserved in all cases in the present study. The initial trauma acted as a stress momentum to the lateral meniscus and may have created a visually occult alteration in the meniscal root apparatus or inner structure. Healing of a bony avulsion of the meniscal root in an extraanatomical position may possibly lead to a lengthening of the physiological circumference resulting in a moderate insufficiency. The degree of dislocation of the fragments in such cases seems to influence the insertion site of the meniscus. The amount of lateral plateau depression and TP widening on the plain anteroposterior radiograph has been proven to be associated with meniscal injury [18]. Occult or unrecognized injury becomes manifest in the long term: MRI in nearly all cases showed some kind of lesion of the lateral meniscus. The severity of the meniscal lesions in our patients was related to the degree of widening of the TP, but not with the severity of the impression of the lateral joint surface. Even in knees with a surgically restored width, the association between the initial widening and the subsequent deterioration of the meniscal condition was demonstrated. Therefore, the status of the meniscus can be attributed to the initial trauma. Gardner et al. [19] reported that significant meniscal tears occurred in up to 90 % of their cases. Preoperative identification of meniscal lesions is desirable because it can change the operative strategy, especially in patients undergoing a minimally invasive approach. The treating surgeon should be aware that a variety of soft tissue injuries are commonly associated with these fractures.

A meta-analysis showed that the incidence of OA after ACL rupture is higher than that after knee fractures [20]. In the present study, no meniscectomies were performed at the time of the index procedure; therefore, no conclusion regarding the relationship between instability or meniscectomy and the incidence of OA can be made. Additionally, cartilage lesions predispose to the development of OA [21], but our data do not allow for any definitive conclusions on this subject.

### Limitations and strengths of the study

The present study serves as an observational MRI study; thus, its main merit is the generation of hypotheses. Our patients were highly selected in terms of the specific implant used for internal fixation and the absence of meniscal and ACL tears; these circumstances do not reflect the actual number of associated pathological conditions in the general clinical setting. No preoperative MRI evaluation of the knee was performed at the time of injury, either because it was unavailable or not part of the preoperative management protocol. We supposed the absence of meniscal and ACL tears at the time of surgery by intraoperative clinical examination and absence of clinical signs of rupture in the first postoperative period by reviewing all available reports.

The clinical skills and diagnostic tools necessary to diagnose these injuries have since improved. Furthermore, we were dependent upon the operation report stating the presence of any type of soft tissue injury.

Measurements of the MRI were performed by an experienced radiologist regarding knee injuries digitally on a workstation. The MRI scans were reviewed and measured once by one radiologist. Therefore, statements to interoberserver and intraobserver reliability of the radiographic measurements cannot be made.

Finally, the study group was too small to generalize the results. However, a major strength of the study is our ability to examine the patients clinically and radiologically after at least 13 years and to give a detailed report of OA and soft tissue injuries.

## Conclusion

All patients showed relevant pathological changes on MRI. A disproportionally high number of structural changes in the medial collateral ligament and ACL were encountered. Late damage to the lateral meniscus was correlated to the initial widening of the TP, but not to the depth of the articular depression.

## Abbreviations

MRI: Magnetic resonance imaging
TPFs: Tibial plateau fractures
OA: Osteoarthritis
KOOS: Knee injury and osteoarthritis outcome score
WORMS: Whole-organ magnetic resonance score
TP: Tibial plateau
RS: Spearman’s rank correlation coefficients
95 % CI: 95 % confidence intervals
ACL: Anterior cruciate ligament
FS-PDw: Fat-suppressed proton-density-weighted

## References

[1] Mink JH, Deutsch AL (1989). Occult cartilage and bone injuries of the knee: detection, classification, and assessment with MR imaging. Radiology.

[2] Fischer SP, Fox JM, Del Pizzo W, Friedman MJ, Snyder SJ, Ferkel RD (1991). Accuracy of diagnoses from magnetic resonance imaging of the knee. A multi-center analysis of one thousand and fourteen patients. J Bone Joint Surg Am.

[3] Reicher MA, Bassett LW, Gold RH (1985). High-resolution magnetic resonance imaging of the knee joint: pathologic correlations. AJR Am J Roentgenol.

[4] Reicher MA, Rauschning W, Gold RH, Bassett LW, Lufkin RB, Glen W (1985). High-resolution magnetic resonance imaging of the knee joint: normal anatomy. AJR Am J Roentgenol.

[5] Foltin E, Fischmeister MF, Wurdinger W (1990). Die Versorgung der Tibiakopffrakturen mit der Gabelplattenach Streli. Ergebnisse einer Nachuntersuchung von 94 Frakturen bei 92 Patienten. Hefte zur Unfallchirurgie.

[6] Fischmeister MF, Schneiderbauer A (2000). Die operative Behandlung von proximalen Tibiafrakturen Typ B und C mit der Gabelplatte. Acta Chir Austriaca.

[7] Mattiassich G, Foltin E, Scheurecker G, Schneiderbauer A, Kropfl A, Fischmeister M (2014). Radiographic and clinical results after surgically treated tibial plateau fractures at three and twenty two years postsurgery. Int Orthop.

[8] Roos EM, Lohmander LS (2003). The Knee injury and Osteoarthritis Outcome Score (KOOS): from joint injury to osteoarthritis. Health Qual Life Outcomes.

[9] Roos EM, Toksvig-Larsen S (2003). Knee injury and Osteoarthritis Outcome Score (KOOS) - validation and comparison to the WOMAC in total knee replacement. Health Qual Life Outcomes.

[10] Peterfy CG, Guermazi A, Zaim S, Tirman PF, Miaux Y, White D, Kothari M, Lu Y, Fye K, Zhao S (2004). Whole-Organ Magnetic Resonance Imaging Score (WORMS) of the knee in osteoarthritis. Osteoarthritis Cartilage.

[11] Crues JV, Mink J, Levy TL, Lotysch M, Stoller DW (1987). Meniscal tears of the knee: accuracy of MR imaging. Radiology.

[12] Cooper DE, Arnoczky SP, Warren RF (1991). Meniscal repair. Clin Sports Med.

[13] Delamarter RB, Hohl M, Hopp E (1990). Ligament injuries associated with tibial plateau fractures. Clin Orthop Relat Res.

[14] Kennedy JC, Bailey WH (1968). Experimental tibial-plateau fractures. Studies of the mechanism and a classification. J Bone Joint Surg Am.

[15] Rademakers MV, Kerkhoffs GM, Sierevelt IN, Raaymakers EL, Marti RK (2007). Operative treatment of 109 tibial plateau fractures: five- to 27-year follow-up results. J Orthop Trauma.

[16] Boszotta H, Helperstorfer W, Kolndorfer G, Prunner K (1993). Long-term results of surgical management of tibial head fractures. Aktuelle Traumatol.

[17] Honkonen SE (1994). Indications for surgical treatment of tibial condyle fractures. Clin Orthop Relat Res.

[18] Durakbasa MO, Kose O, Ermis MN, Demirtas A, Gunday S, Islam C (2013). Measurement of lateral plateau depression and lateral plateau widening in a Schatzker type II fracture can predict a lateral meniscal injury. Knee Surg Sports Traumatol Arthrosc.

[19] Gardner MJ, Yacoubian S, Geller D, Suk M, Mintz D, Potter H, Helfet DL, Lorich DG (2005). The incidence of soft tissue injury in operative tibial plateau fractures: a magnetic resonance imaging analysis of 103 patients. J Orthop Trauma.

[20] Spahn G, Schiele R, Hofmann GO, Schiltenwolf M, Grifka J, Vaitl T, Scheidler S, Liebers F, Seidler S, Klinger HM (2011). The Relative Risk of Knee Osteoarthritis after Knee Injuries – Results of a Metaanalysis. Phys Med Rehab Kuror.

[21] Heijink A, Gomoll AH, Madry H, Drobnic M, Filardo G, Espregueira-Mendes J, Van Dijk CN (2012). Biomechanical considerations in the pathogenesis of osteoarthritis of the knee. Knee Surg Sports Traumatol Arthrosc.

